# Spatial heterogeneity of malaria in Indian reserves of Southwestern Amazonia, Brazil

**DOI:** 10.1186/1476-072X-7-55

**Published:** 2008-11-03

**Authors:** Reinaldo Souza-Santos, Maurício VG de Oliveira, Ana Lúcia Escobar, Ricardo Ventura Santos, Carlos EA Coimbra

**Affiliations:** 1 Escola Nacional de Saúde Pública, Fundação Oswaldo Cruz, Leopoldo Bulhões 1480, Rio de Janeiro, RJ 21041-210, Brazil; 2 Centro de Estudos em Saúde do Índio de Rondônia, Universidade Federal de Rondônia, Rodovia BR-364 Km 9, Porto Velho, RO 78900-000, Brazil; 3 Departamento de Antropologia, Museu Nacional, Quinta da Boa Vista s/n, Rio de Janeiro, RJ 20940-040, Brazil

## Abstract

**Background:**

Malaria constitutes a major cause of morbidity in the Brazilian Amazon where an estimated 6 million people are considered at high risk of transmission. Indigenous peoples in the Amazon are particularly vulnerable to potentially epidemic disease such as malaria; notwithstanding, very little is known about the epidemiology of malaria in Indian reservations of the region. The aim of this paper is to present a spatial analysis of malaria cases over a four-year time period (2003–2006) among indigenous peoples of the Brazilian State of Rondônia, southwestern Amazon, by using passive morbidity data (results from Giemsa-stained thick blood smears) gathered from the National Malaria Epidemiologic Surveillance System databank.

**Results:**

A total of 4,160 cases of malaria were recorded in 14 Indian reserves in the State of Rondônia between 2003 and 2006. In six reservations no cases of malaria were reported in the period. Overall, *P. vivax *accounted for 76.18 of malaria cases reported in the indigenous population of Rondônia. The *P. vivax/P. falciparum *ratio for the period was 3.78. Two reserves accounted for over half of the cases reported for the total indigenous population in the period – Roosevelt and Pacaas Novas – with a total of 1,646 (39.57%) and 1,145 (27.52%) cases, respectively. Kernel mapping of malaria mean Annual Parasite Index – API according to indigenous reserves and environmental zones revealed a heterogeneous pattern of disease distribution, with one clear area of high risk of transmission comprising reservations of west Rondônia along the Guaporé-Madeira River basins, and another high risk area to the east, on the Roosevelt reserve.

**Conclusion:**

By means of *kernel *mapping, it was shown that malaria risk varies widely between Indian reserves and environmental zones defined on the basis of predominant ecologic characteristics and land use patterns observed in the southwestern Brazilian Amazon. The geographical approach in this paper helped to determine where the greatest needs lie for more intensively focused malaria control activities in Indian reserves in the region. It also provided a reference to assess the effectiveness of control measures that have been put in place by Brazilian public health authorities.

## Background

Malaria is recognized as a major cause of morbidity in Brazil. The Amazon region, which represents approximately 60% of the national territory, accounts for nearly 99% of all cases in the country. During the 1990s more than 600,000 cases were reported annually [1,2]. In spite of the relative success of efforts to control malaria over the last decades, the disease remains an important cause of morbidity in the Brazilian Amazon, with an estimated 6 million people considered at high risk of malaria transmission [2].

An important feature of current malaria epidemiology in the Brazilian Amazon is its focal pattern: the spatial distribution of cases is not homogeneous throughout the region, but localized, depending upon the interplay between a number of socio-demographic and environmental factors [3,4]. Thus, rural populations living on the banks of major rivers, those in areas that underwent extensive deforestation due to development projects (such as agricultural colonization, cattle ranching or power-plants), or others who live in areas under the influence of mining activities face different risks of disease transmission [5-9].

Generally speaking, indigenous peoples in the Amazon are highly exposed to *Plasmodium *infection [10]. Nonetheless, it is difficult to make generalizations about malaria epidemiology for the whole indigenous population in the region, since incidence rates vary widely, ranging from Indian reserves that are almost malaria-free or where chronic asymptomatic infection predominates, to reservations where high incidence of disease and mortality prevails [11-14]. Even within the same ethnic group, different villages may present, on the one hand, a hyperendemic and stable transmission pattern and, on the other hand, mesoendemic and unstable transmission, as was the case of the Yanomami, who live on the Brazilian-Venezuelan border [15].

Spatially targeted interventions for malaria control are recognized as a good approach to low-cost disease control [1,16,17]. In Amazonia, most recent spatial analyses of malaria have dealt almost exclusively with populations living in areas of agricultural colonization or mining sites [4,18-21]. Despite their greater vulnerability to infectious diseases that are potentially epidemic, such as malaria [10,22], indigenous peoples in Amazonia have not been considered in ongoing malaria control-oriented epidemiologic research in the region.

The objective of this paper is to present a spatial analysis of malaria cases over a four-year time period (2003–2006) among indigenous peoples in the Brazilian State of Rondônia, in the southwestern region of the Amazon. This State has been at the center of international debate about some of the most striking consequences of human occupation of Amazonia in recent decades [23-25]. Extensive malaria research has been carried out in the region under different epidemiologic contexts – hypoendemic among riverine populations and epidemic among new settlers [1,7,21,26-28]. This paper aims at contributing to include indigenous peoples in the ongoing debates about malaria epidemiology, control strategies and surveillance systems focused on spatially-targeted areas or populations in the Amazon.

## Population and methods

### Setting

The State of Rondônia, with an area of 238,512.8 km^2^, is situated in the southwestern portion of the Brazilian Amazon. From an ecological perspective, the region is highly diversified and includes tropical ecosystems that range from open grasslands and savannas to upland forests and floodplains. Mean annual temperatures in Rondônia vary between 24 and 26°C, while annual rainfall ranges from 2,500 mm in the north to 1,750 mm on the southern border. The rainy season peaks between January and March, while the driest months are between June and August. The mean pluviometric difference between the rainiest and the driest months is around 350 mm, thus presenting one of the highest pluviometric amplitudes in Brazil [29].

During the twentieth century, Rondônia experienced several demographic-economic frontier cycles, including rubber extraction, gold-mining, agricultural colonization, timber extraction, cattle ranching, and large-scale development projects (including the construction of hydroelectric power-plants and federal highways) [25,30]. Combined, these activities have had a profound impact on the environment, directly influencing the dynamics of malaria transmission in the region [31]. In some instances, migration and environmental disruptions triggered alarming epidemics, as was the case in the gold-mining sites along the Madeira River in the 1980s [24,32]. In other places, the expansion of land clearings for agriculture and pasture was so intense that malaria transmission was almost curtailed due to the overall transformation of the landscape and the elimination of anopheline breeding places [5]. Indigenous peoples were directly affected by these cycles, and suffered the worst possible consequences from land invasions, epidemics and environmental contamination from mercury that came from gold smelting [22,33,34].

The patterns of human settlement and land use have varied widely within the State of Rondônia and they appear to correlate with different malaria epidemiologic profiles observed across time and space. Land clearings for pasture and agriculture were most intense in the municipalities closest to the BR-364, a major highway that crosses the State from north to south. For example, in the municipality of Ji-Paraná, an important center for agribusiness and small industries, intense forest clearing extends at least 50 km or more from the edges of the highway [35]. To the west, along the Guaporé-Madeira River basins, the situation is different and more typical of Amazonian floodplains. The human population is mostly scattered across small towns and villages along the main rivers and its tributaries. In this region, anophelines are ubiquitous and malaria is endemic. Dividing these two poles in a northwesterly-southeasterly direction in central Rondônia is the Pacaas-Novos plateau. Colonization and agricultural activities in this area are limited, since most of it is under the protection of national forests or inhabited by relatively small indigenous groups.

### Environmental Classification of Indian Reserves in Rondônia

**Figure 1:**
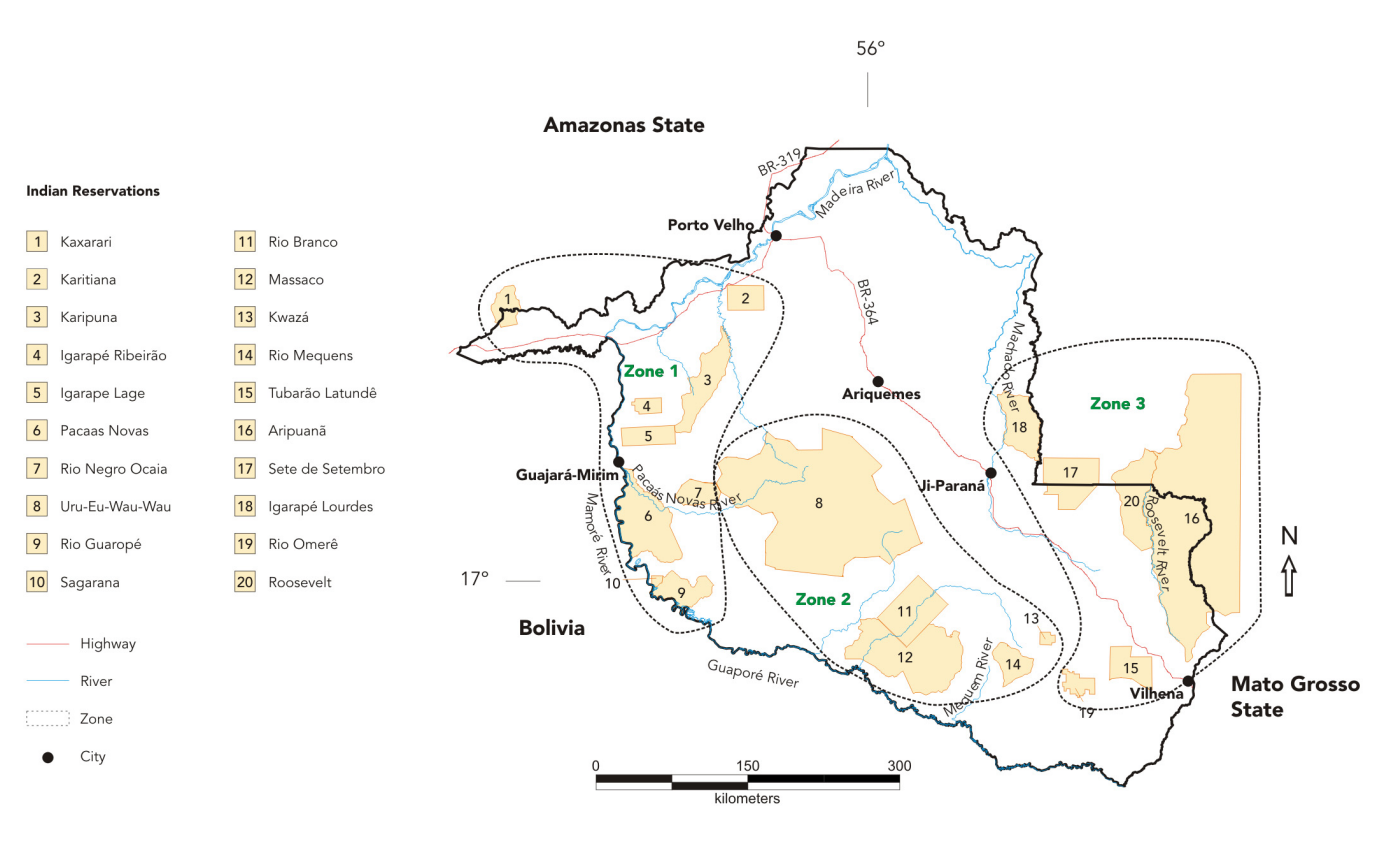
Indigenous reserves and environmental zone of Rondônia State, Brazilian Amazon.

The indigenous population of the State of Rondônia comprises 16 ethnic groups and totals approximately 7,700 people, according to the Brazilian National Health Foundation – FUNASA  (Table 1). All Indian reserves situated within State boundaries were included in the study. This makes a total of 20 reserves, three of which (Aripuanã, Roosevelt, and Sete de Setembro) extend beyond Rondônia's borders to the east and into the State of Mato Grosso, and one (Kaxarari) that crosses the northwest border into the State of Amazonas. For the purposes of data analysis, the state's Indian reserves were grouped into three Zones based on their predominant environmental/ecologic characteristics and pattern of land use in the area (Figure 1).

**Table 1:** Indian reserves by zone and population size, Rondônia State, Brazil

**Indian Reserves by zones of stratification**	**Population size**	
	**n**	**%**
**Zone 1 – *Guaporé/Madeira River basin***		
Igarapé Lage	309	4.01
Igarapé Ribeirão	236	3.06
Karipuna	25	0.32
Karitiana	228	2.96
Kaxarari	273	3.54
Pacaas Novas	2546	33.03
Rio Guaporé	89	1.15
Rio Negro Ocaia	456	5.92
Sagarana	261	3.39
**Zone 2 – *Pacaas-Novos Plateau***		
Rio Branco	539	6.99
Uru-Eu-Wau-Wau	226	2.93
Massaco	n/a	n/a
Kwazá	28	0.36
Rio Mequens	21	0.27
**Zone 3 – *Ji-Paraná/Aripuanã River basins***		
Igarapé Lourdes	442	5.73
Aripuanã	360	4.67
Roosevelt	659	8.55
Rio Omerê	11	0.14
Sete de Setembro	829	10.75
Tubarão Latundê	171	2.22

**Total**	7709	100.00

• Zone 1 – *Guaporé/Madeira River basin*: this Zone includes eight indigenous reserves situated along the right banks of the Guaporé, Mamoré, and Madeira rivers, including their major tributaries (Branco, Pacaas-Novos, Mequens, and São Miguel rivers), and one reserve located on the northwestern side of the Madeira River – the Kaxarari. Ecologically, this Zone is dominated by typical Amazonian white-water flood-plains [36], where breeding places for anophelines often abound. This region first opened up to permanent contact with wider Brazilian society through the Madeira-Mamoré railway that was inaugurated in the early twentieth century. It has since remained relatively spared from the mainstream development projects that have been carried out in Rondônia since the 1970s, and the expansion of agribusinesses and the urban network have been kept to a minimum. However, throughout the first half of the last century indigenous peoples in this zone were hit hard by first-contact epidemics of measles, malaria, and tuberculosis. The indigenous population in this zone is estimated to be 4,423, made up of more than ten different ethnic groups, and representing 57.4% of the total Indian population of the State. At present, malaria is endemic along the banks of the Guaporé, Mamoré, and Madeira rivers and there is a high prevalence of chronic carriers of *P. vivax *among populations living in these areas [1,27,28,37].

• Zone 2 – *Pacaas-Novos plateau*: this Zone basically falls within the limits of the Uru-Eu-Wau-Wau Indian reserve and the Jaru National Forest. This region is distinguished from the rest of the State in that it includes elevated areas ranging from 500 to 1,000 m and vegetation coverage that is dominated by grasslands and savannas. Gallery forests accompany the course of the myriad of small streams that flow to the west or east of the plateau, feeding into the Guaporé-Madeira to the west or Ji-Paraná river systems to the east. Despite the region's relative isolation from recent economic frontiers, it has been under pressure in recent years from the combined activities of illegal loggers and small-scale farmers seeking new sources of timber and agricultural land [35]. Land squatters and loggers constitute important links in the epidemiology of malaria in this Zone, not only because of their likelihood of introducing strains of *P. vivax *and *P. falciparum *but also because of the environmental impacts of their activities that favor the breeding of anopheline mosquitoes [8,32]. The indigenous population totals approximately 814 individuals (10.6% of the State) and is basically made up of small Tupi-speaking groups that inhabit the Uru-Eu-Wau-Wau Reserve. There is no previous information about malaria available for this region of Rondônia.

• Zone 3 – *Ji-Paraná/Aripuanã River basins*: This region consists of typical Amazonian interfluvial upland forests [36], covered by dense vegetation with limited penetration of sunlight and relatively clear understory, crisscrossed by small to mid-size rivers and streams, all feeding into the Ji-Paraná or Aripuanã/Roosevelt rivers. The completion of the BR-364 highway in the late 1960s allowed thousands of colonists to move into the area and to settle in small plots, clearing land for coffee and pasture [25,30]. Once endemic, malaria incidence in most municipalities in this zone has declined dramatically over the last two decades and, in some cases, has been practically eradicated – probably reflecting the extent to which the territory has been transformed over this period [38]. More recently, there has been a trend towards land concentration, as small-scale colonization has been replaced by cattle ranching. The indigenous population in this zone totals some 2,472 individuals (32.1% of the State), mostly Tupi-speakers, who inhabit five reserves. An important aspect is that, since early 2000, diamond prospecting in the Cinta-Larga reserve has attracted hundreds of miners from all over the Amazon region. This is potentially a major factor in triggering malaria epidemics in the region.

### Data and Analysis

This study relied on passive morbidity data from 2003 to 2006 gathered on-line through the National Malaria Epidemiologic Surveillance System (SIVEP-Malaria)  that is maintained by the Brazilian Ministry of Health. Passive case detection in Brazil relies on a network of public health units that provide free diagnosis (Giemsa-stained thick blood smears) and standard treatment for subjects seeking care for malaria. We are aware that standard Giemsa-stained thick blood smears are not sensitive enough to detect all clinical cases of malaria, especially when parasitemia is low. Despite this well-known limitation, it remains the standard technique used in most endemic countries because of its low cost and practicability. Therefore, malaria cases herein reported are likely to be underestimated. The results from microscopic exams are classified by SIVEP according to the "probable" place of infection (e.g., village, municipality). For the purposes of this study, SIVEP records were grouped by the Indian reserve where the subject's village is situated, stratified by sex, species of *Plasmodium *(*vivax *and/or *falciparum*), and year of notification. The Annual Parasite Index – API (total number of positive blood slides per total population) was calculated for each Indian reserve. The mean API for the period 2003–2006 for each reserve was calculated by summing up the API of each year divided by four [18]. In order to conduct a GIS-based analysis of the spatial distribution of malaria, a reserve-level polygon map was developed for the state. Cases of malaria were matched to the reserve-level layer using the TerraView^® ^software, version 3.1.4 .

*Kernel *mapping was the method used to analyze the spatial patterns of mean API distribution and to identify high risk areas (hot spots) for malaria [39]. Accordingly, a *kernel *function was superimposed over a grid of locations (Indian reserves) and the (distance-weighted) density of point events was estimated for each Zone, with the degree of smoothing controlled by the *kernel *bandwidth. We opted for a *kernel *map with 150 columns on the events and with a quartic function algorithm with a radius of 90 km. Through this method we aimed to show how malaria risk (accessed by mean API) is spatially distributed throughout Rondônia state, thus providing a useful visual indication of the variation in the intensity of malaria transmission in Indian reserves. We used the terminology low, medium, and high risk for the analysis of the spatial patterns of malaria risk for the Indian reserves, considering the centroid of each reserve. TerraView^® ^software, version 3.1.4, was used to calculate the mean intensity of API in Indian reserves divided according to the three environmental Zones described above.

## Results

A total of 4,160 cases of malaria were recorded in 14 Indian reserves in the state of Rondônia between 2003 and 2006 (Table 2). No records of malaria were obtained for the populations living at the Massaco, Rio Mekens, Rio Negro Ocaia, Rio Omerê, Aripuanã, and Kwazá reserves.

**Table 2:** Malaria cases (positive blood smears) and Annual Parasite Index (API) in Indian reserves and environmental zones of Rondônia State, Brazilian Amazon.

**Indian reserve**	**2003**		**2004**		**2005**		**2006**		**Mean**
	**Positive**	**API**	**Positive**	**API**	**Positive**	**API**	**Positive**	**API**	**API/1000**
Zone 1 – Guaporé/Madeira River basin*									
Igarapé Lage	54 15.30%	174.76	33 2.09%	106.80	61 7.96%	197.41	141 9.66%	456.31	233.82
Igarapé Ribeirão	13 3.68%	55.08	39 2.47%	165.25	59 7.70%	250.00	72 4.93%	305.08	193.86
Karipuna	21 5.95%	840.00	10 0.63%	400.00	0 0.00%	0.00	4 0.27%	160.00	350.00
Karitiana	34 9.63%	149.12	37 2.34%	162.28	23 3.00%	100.88	42 2.88%	184.21	149.12
Kaxarary	111 31.44%	406.59	152 9.61%	556.78	91 11.88%	333.33	54 3.70%	197.80	373.63
Pacaas Novas	78 22.10%	30.64	269 17.01%	105.66	309 40.34%	121.37	489 33.49%	192.07	112.43
Rio Guaporé	1 0.28%	11.24	0 0.00%	0.00	1 0.13%	11.24	10 0.68%	112.36	33.71
Sagarana	1 0.28%	3.83	6 0.38%	22.99	4 0.52%	15.33	2 0.14%	7.66	12.45
**Subtotal**	**313**	**78.90**	**546**	**137.64**	**548**	**138.14**	**814**	**205.19**	**139.97**

Zone 2 – Pacaas-Novos plateau**									
Rio Branco	3 0.85%	5.57	5 0.32%	9.28	103 13.45%	191.09	44 3.01%	81.63	71.89
Uru-Eu-Wau-au	12 3.40%	53.10	7 0.44%	30.97	12 1.57%	53.10	31 2.12%	137.17	68.58
**Subtotal**	**15**	**19.61**	**12**	**15.69**	**115**	**150.33**	**75**	**98.04**	**70.92**

Zone 3 – Ji-Paraná/Aripuanã River basins***									
Igarapé Lourdes	16 4.53%	36.20	7 0.44%	15.84	7 0.91%	15.84	9 0.62%	20.36	22.06
Roosevelt	4 1.13%	6.07	1001 63.31%	1518.97	92 12.01%	139.61	549 37.60%	833.08	624.43
Sete de Setembro	4 1.13%	4.83	13 0.82%	15.68	3 0.39%	3.62	12 0.82%	14.48	9.65
Tubarão Latundê	1 0.28%	5.85	2 0.13%	11.70	1 0.13%	5.85	1 0.07%	5.85	7.31

**Subtotal**	**25**	**11.90**	**1023**	**486.91**	**103**	**49.02**	**571**	**271.78**	**204.90**

**Total**	**353 100%**	**51.66**	**1581 100%**	**231.38**	**766 100%**	**112.10**	**1460 100%**	**213.67**	**152.20**

* No cases of malaria were recorded in Rio Negro Ocaia reserves (Zone 1).

** No cases of malaria were recorded in Kwazá, Massaco and Rio Mequens reserves (Zone 2).

*** No cases of malaria were recorded in Aripuanã and Rio Omerê, reserves (Zone 3).

The *P. vivax/P. falciparum *ratio for the period was 3.78. A trend showing an increase in species ratio in favor of *P. vivax *was noted throughout the period (1.79 in 2003; 3.09 in 2004; 4.47 in 2005; 5.63 in 2006). Only in 2003 we observed more *P. falciparum *then *P. vivax *in Igarapé Lourdes, Kaxarary, Rio Branco and Roosevelt (ratios of 0.60, 0.73, 0.50, and 0.33 respectively). Overall, *P. vivax *accounted for 76.2% of malaria cases reported in the indigenous population of Rondônia. Mixed *P. vivax *and *P. falciparum *infections were nearly inexistent (only 3.6%). The remaining cases (20.2%) were caused by *P. falciparum*. *P. malariae *was not recorded. Two reserves accounted for over half of the cases reported for the total indigenous population in the period – Roosevelt and Pacaas Novas reserves, with a total of 1,646 (39.57%) and 1,145 (27.52%) malaria cases, respectively.

Of the nine reserves situated in Zone 1, six showed mean API for the period of more than 100/1,000 inhabitants (Table 2). The mean API at Karipuna and Kaxarary reserves were 350 and 373.63/1,000 inhabitants, respectively. Most Zone 1 reserves presented API of more than 100/1,000 inhabitants and a rising trend between 2003 and 2006. The highest API observed for Zone 2 Indian reserves was between 68 and 71/1,000 inhabitants, reported for Uru-Eu-Wau-Wau and Rio Branco reserves. The highest API in the State was observed in Zone 3, at the Roosevelt Reserve – 624.43/1,000 inhabitants on average for the period. Most other reserves in this zone presented relatively low malaria risk, with API less than or equal to 22. The annual variation in API reported for the Roosevelt reserve was strikingly different from the pattern observed in other Indians reserves of Rondônia, with peaks observed in 2004 and 2006. A comparison of the average API between zones showed that the highest API was reported in Zone 3 (204.9), a result that was directly influenced by the situation at the Roosevelt reserve. Mean API in Zones 1 and 2 were 140.0 and 70.9, respectively (Table 2).

In all reserves, most cases of malaria in the period were reported among male subjects (Table 3).

**Table 3:** Malaria cases (positive blood smears) by sex and Indian reserves, State of Rondônia, Brazilian Amazon, 2003–2006.

**Indian reserve**	**2003**		**2004**		**2005**		**2006**		**Total**		
	**Male**	**Female**	**Male**	**Female**	**Male**	**Female**	**Male**	**Female**	**Male**	**Female**	**Total**
Zone 1 – Guaporé/Madeira River basin*											
Igarapé Lage	23	31	10	23	31	30	84	57	**148**	**141**	**289**
Igarapé Ribeirão	10	3	23	16	28	31	36	36	**97**	**86**	**183**
Karipuna	17	4	7	3	0	0	4	0	**28**	**7**	**35**
Karitiana	18	16	22	15	15	8	23	19	**78**	**58**	**136**
Kaxarary	62	49	84	68	51	40	31	23	**228**	**180**	**408**
Pacaas Novos	38	40	163	106	184	125	288	201	**673**	**472**	**1145**
Rio Guaporé	1	0	0	0	0	1	1	9	**2**	**10**	**12**
Sagarana	1	0	3	3	2	2	0	2	**6**	**7**	**13**
Zone 2 – Pacaas-Novos plateau**											
Rio Branco	2	1	5	0	55	48	30	14	**92**	**63**	**155**
Uru-Eu-Wau-Wau	4	2	5	2	11	1	19	6	**39**	**11**	**50**^+^
Zone 3 – Ji-Paraná/Aripuanã River basins***											
Igarapé Lourdes	12	4	5	2	4	3	5	3	**26**	**12**	**38**^++^
Roosevelt	3	1	762	239	78	14	449	100	**1292**	**354**	**1646**
Sete de Setembro	4	0	9	4	3	0	10	2	**26**	**6**	**32**
Tubarão Latundê	1	0	2	0	1	0	0	0	**4**	**0**	**4**^+++^

**Total**	**196**	**151**	**1100**	**481**	**463**	**303**	**980**	**472**	**2739**	**1407**	**4146**

* No cases of malaria were recorded in Rio Negro Ocaia reserves (Zone 1).

** No cases of malaria were recorded in Kwazá, Massaco and Rio Mequens reserves (Zone 2).

*** No cases of malaria were recorded in Aripuanã and Rio Omerê, reserves (Zone 3).

+ Missing records for sex variable: 2003 (6); 2006 (6).

++ Missing records for sex variable: 2006 (1).

+++ Missing records for sex variable: 2006 (1).

**Figure 2:**
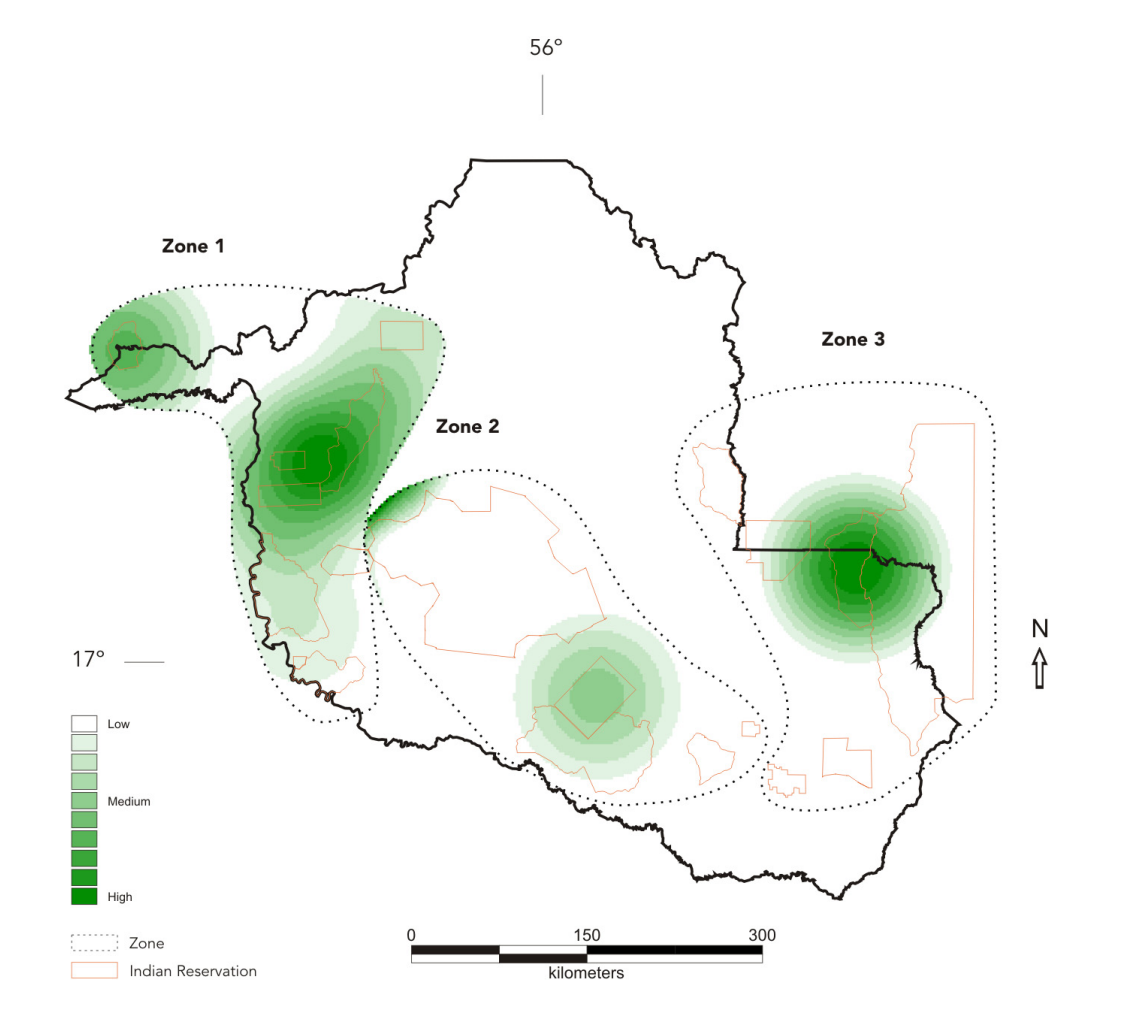
Kernel estimation of malaria mean API (2003–2006), according to indigenous reserves and environmental zones, Rondônia, Brazil.

Figure 2 shows the relative density of mean API distribution in Indian reserves in the three zones. Upon visual comparison of the images it appears that the density of mean API is more focused towards reserves situated in Zone 1, with a clear high risk area for malaria comprising the Karipuna, Igarapé Ribeirão, and Igarapé Lage reserves. Another area of high risk can be seen in Zone 3, with a malaria hot spot in the Roosevelt reserve and neighboring areas. Zone 2 only shows areas of intermediate risk of malaria, limited to the Rio Branco and Uru-Eu-Wau-Wau reserves. With few exceptions, most other reserves in Zones 2 and 3 showed very low risk of malaria.

## Discussion

The results of this study show that malaria epidemiology in indigenous reserves of Rondônia exhibit a highly unstable and heterogeneous profile. A notable finding is the significant difference in API between the zones into which Rondônia Indian reserves were divided. These zones – floodplains to the west (Zone 1), upland savannas and springs in the central plateau (Zone 2), and upland interfluvial forest to the east (Zone 3) – were defined on the basis of predominant ecological factors and patterns of land use. Our findings indicate that human ecology and landscape transformations may have major short- and long-term impacts on malaria epidemiology in the indigenous reserves of Rondônia, either triggering serious outbreaks or, in other contexts, nearly ceasing transmission, as has been documented in areas where extensive deforestation for agribusiness expansion took place.

Despite an important trend indicating a decrease in malaria rates in the four-year-period of this study (approximately 40% of indigenous reserves were nearly malaria-free or completely free; API < 10), this result should not be seen as definitive. The environmental capacity for malaria transmission in Rondônia is high. The disease can resurge and rapidly increase its rate of transmission after years of apparent "eradication" in response to landscape changes brought by anthropogenic interventions – the short-term impact of diamond prospecting on malaria epidemiology at the Roosevelt reserve after years of control is a case in point.

*Anopheles darlingi *is the major malaria vector in Rondônia as well as in most Brazilian Amazonia [31,40-42]. This species is highly anthropophilic and exhibits a predominantly exophilic behavior, i.e., it either feeds on humans when individuals are carrying out any kind of outdoor activity, or it enters houses to feed and leaves shortly after a blood meal to rest on nearby vegetation. *A. darlingi *breeds profusely in sunlit pools of water in areas of natural floodplains (for example the western Rondônia Guaporé-Madeira River system – Zone 1) or in recently deforested areas, where drainage has been disturbed (e.g. mining sites – Zone 3).

Previous studies carried out in west Rondônia (Zone 1), a region that is dominated by the hydrographic systems of the Guaporé-Madeira rivers, show the historic continuity and year-round transmission of malaria in this area, characterized by high prevalence rates of asymptomatic *P. vivax *infection in the rural population [14,27,28]. Both indigenous and non-indigenous ('*caboclos*') villages in this Zone are typically situated close to rivers and streams, where anophelines abound and represent a constant nuisance. Houses are usually made of wood boards and palm thatch that allow numerous crevices through which mosquitoes may freely penetrate [14]. Most reserves situated in Zone 1 (API > 100/1,000) are inhabited by Warí' (Pakaanóva) Indians, the largest indigenous group in Rondônia. The Wari' spend part of the year tending their gardens far from their villages and in hunting and gathering treks. Night fishing is carried out by men year-round. Inter-village visits for social and commercial exchanges are common. These behaviors probably represent important factors in the dissemination and maintenance of malaria transmission in Indian reserves of the Guaporé-Madeira system, especially among the Wari' people and their neighbors [14].

The situation of malaria in the indigenous reserves situated in eastern Rondônia (Zone 3) suggests a declining trend in the number of cases reported with one exception – the Roosevelt reserve inhabited by Cinta-Larga Indians. The mean API reported for Indian reserves in this Zone was less than 20/1,000; with single digits in most areas. However, the apparently stable epidemiologic situation changed dramatically in 2004. That was when a diamond mining rush attracted over 2,000 illegal miners into the reserve [43]. The miners caused extensive deforestation and disruption in river beds as digging and washing of gravel proceeded rapidly. This frenetic intervention on the environment coupled with the migration of miners, many of whom were already infected with plasmodia, led to a malaria epidemic on a scale that was unheard of among the indigenous reserves of Rondônia since the early 1980s. Just before diamond mining began, malaria transmission among the Cinta-Larga Indians from the Roosevelt reserve was nearly interrupted (API = 4/1,000). This number jumped to 1,518/1,000 in 2004, when Brazilian authorities managed to achieve some control of the social upheaval that had arisen within the boundaries of the reserve by the end of the year, removing the miners and shutting-down the diamond hot-spots in the reserve. The relative control of mining activities in the reserve is reflected in the drastic drop in the number of cases in the following year, a situation that did not last long, however, since miners partially returned by the end of 2005, raising the API in the reserve to 833/1,000 in 2006. The explosive nature of malaria in mining sites throughout the Amazon is well documented. During the study period, Cinta-Larga Indian reserves, particularly Roosevelt and Aripuanã, were under intense pressure from miners. Gold mining sites had been established relatively close to these reserves and malaria epidemics were also a serious problem in these areas [43,44].

Indian reserves in Central Rondônia (Zone 2) showed intermediate API, compared to what was observed in Zones 1 and 3. Ecologically speaking, Zone 2 does not seem particularly prone to sustain important anopheline breeding places. Malaria transmission in the area appears to be directly related to illegal logging activities in Indian reserves.

In this paper, a map showing the spatial variation of malaria risk in Indian reserves of southwestern Brazilian Amazon was generated using *kernel *estimates of the mean Annual Parasite Index. The map is a first attempt towards a spatial analysis of malaria risk in Indian reserves of Brazilian Amazonia. Despite their vulnerability to malaria epidemics, indigenous peoples in Brazil had been subject to very few epidemiologic studies, thus precluding any possible generalization at the regional level. The map shows that malaria risk varies widely between reserves and environmental zones defined on the basis of predominant ecologic characteristics and land use patterns. The analysis points to high risk areas in reserves located along river banks to the west of Rondônia known for its high prevalence of asymptomatic *Plasmodium*-carriers and for its ubiquitous, year-round anopheline population. It also highlights the important role played by mining activities in triggering malaria epidemics within Indian reserves, a situation that clearly marked one particular reserve in Zone 3, despite an overall downward trend in malaria cases in east Rondônia.

## Conclusion

Malaria control activities in Indian reserves of Amazonia are limited to house spraying with insecticide at irregular intervals and treatment of individuals with a positive thick blood smear. So far, insecticide treated nets or other strategies have not been systematically used in indigenous communities. As is widely known, malaria epidemiology is very complex and is dependent upon environmental, socioeconomic, and human ecological factors, in addition to local vector fauna. Control programs based on assumptions that are not evidence-based are prone to waste resources and be of limited effectiveness to populations at risk.

By means of *kernel *mapping, this paper has demonstrated the heterogeneous pattern of malaria transmission risk in Indian reserves in the southwestern Brazilian Amazon. It is expected that the geographical approach adopted in this paper will assist public health workers determine where greatest needs lie for more intensively focused malaria control activities in Indian reserves, therefore contributing to the critical appraisal of control measures that have been put in place by Brazilian health authorities.

## Competing interests

The authors declare that they have no competing interests.

## Contributors

RSS, MVGO and CEAC worked in all phases of the article. ALE and RVS collaborated in the data analysis and interpretation. All authors reviewed previous drafts and approved the final version submitted for publication.
